# Frequency and Predictors of Conversion From Laparoscopic to Open Cholecystectomy: A Single-Center Observational Study

**DOI:** 10.7759/cureus.76327

**Published:** 2024-12-24

**Authors:** Gohar Ali, Muhammad Zeb, Almas Khattak, Rashid Khan, Muhammmad Kashif Dawar, Khizer Zaman, Nauman Ul Mulk, Junaid Khan, Shakir Ullah

**Affiliations:** 1 General Surgery, Hayatabad Medical Complex Peshawar, Peshawar, PAK; 2 Surgery, Hayatabad Medical Complex Peshawar, Peshawar, PAK; 3 Orthopedics, Khyber Teaching Hospital, Peshawar, PAK

**Keywords:** conversion, difficult laparoscopic cholecystectomy, laparoscopic cholecystectomy, laparoscopic cholecystectomy converted to open cholecystectomy, risk factors

## Abstract

Objective: The study aimed to investigate the rate of conversion from laparoscopic cholecystectomy (LC) to open cholecystectomy (OC) in our population and determine the potential risk factors associated with it. Understanding these factors helps surgeons predict complex cases and plan surgeries, reducing patient risks and improving outcomes.

Methodology: A cross-sectional observational study was conducted from June 1, 2022, to May 31, 2023, at Hayatabad Medical Complex, Peshawar, on 349 patients undergoing elective LC. Data on demographics, clinical history, laboratory values, and imaging findings were recorded using a standardized proforma. Intraoperative findings, surgical outcomes, and complications were noted, with statistical analysis performed using IBM SPSS Statistics for Windows, Version 23.0 (Released 2015; IBM Corp., Armonk, New York, United States). Chi-squared, Mann-Whitney U, and logistic regression tests assessed the associations and risk factors of conversion to open surgery.

Results: The rate of conversion from LC to OC was found to be 13 (3.7%). The multivariate analysis revealed several significant risk factors associated with the conversion. These included male gender, history of jaundice due to gallbladder stones, history of pancreatitis, gallbladder wall thickness greater than 3 mm, white blood cell counts greater than 1000 per microliter of blood, difficulty in handling the gallbladder with instruments intraoperatively, and dense adhesions with surrounding tissues (p<0.05). The risk factor summation pyramid showed a sensitivity of 84.6% and a specificity of 63.8% in predicting the likelihood of conversion, emphasizing the importance of considering each risk factor individually.

Conclusion: The rate of conversion to OC was 3.7%. Factors such as male gender, history of jaundice due to gallbladder stones, history of pancreatitis, thick gallbladder wall, high white blood cell count, difficulty in handling the gallbladder, and dense adhesions with surrounding tissues were significantly associated with conversion to OC.

## Introduction

The prevalence of gallstone disease is influenced by various factors, including age, gender, ethnicity, and geographical location. Additionally, the westernization of diet and changes in socioeconomic status have been linked to an increase in the prevalence of gallstone disease [1,2]. The 4Fs (female gender, fertility, being overweight or having a high-fat diet, and reaching the age of 40) have been recognized since early times as common risk factors for cholelithiasis and are still significant today [3]. In Pakistan, the prevalence of gallstones is 3.07% [4].

According to the Mayo Clinic and the Tokyo criteria, abdominal ultrasound, recommended for its accuracy and cost-effectiveness, is often used to diagnose gallbladder stones and their complications [5,6]. Laparoscopic cholecystectomy (LC) is the preferred treatment for benign gallbladder disease and is considered safe and effective for most symptomatic gallstones, as concluded by the National Institute of Health (NIH) conference in 1993 [1,7,8].

LC has been shown to have several advantages over conventional cholecystectomy, including a lower rate of complications, less postoperative pain, quicker return of bowel function, better cosmetic outcomes, faster recovery, and shorter hospital stays [1,9-11]. However, there are several factors that may necessitate the conversion from LC to open cholecystectomy (OC), such as demographic characteristics, clinical and paraclinical features, as well as intraoperative incidents. These factors need to be taken into consideration to ensure a safe and successful completion of the procedure, even if it requires more time than initially anticipated [10-12].

The conversion rate from LC to OC due to different risk factors ranges from 2% to 15%, according to published studies [9,13]. A recent study in Pakistan reported a conversion rate of 7.78% [2]. In addition, OC is also associated with complications like bile duct injury, intraoperative bleeding, requiring reoperation and transfusion, and even death of the patient [13].

The study's objective was to determine the rate of conversion from LC to OC at a single center and develop a risk prediction model for patients with gallbladder disease who required surgery. The model would aid in surgical planning and decision-making. Experienced surgeons have a lower complication rate than trainee surgeons. Additionally, the prediction model can assist with preparing patients, scheduling surgery, reducing procedure-related costs, and overcoming constraints in developing countries. 

## Materials and methods

A cross-sectional observational study was conducted on patients who required elective LC for indications, i.e., acute calculus cholecystitis, chronic cholecystitis, and gallbladder perforation, from June 1, 2022, to May 31, 2023, at the General Surgery Department, Hayatabad Medical Complex Peshawar, Peshawar, Pakistan. In accordance with the Declaration of Helsinki, the research was approved by the Ethical Review Board of Hayatabad Medical Complex Peshawar (approval number: HMC-QAD-F-1273). Also, the work has been reported in line with the Strengthening the Reporting of Cohort, Cross-Sectional, and Case-Control Studies in Surgery (STROCSS) criteria [14].

After strict inclusion criteria, 349 patients were included in the study. Forty-three patients were excluded on the basis of exclusion criteria. Written and informed consent was taken from each patient. All the patients were planned for elective LC. Under a standardized proforma, patients' preoperative, intraoperative, and postoperative data were collected. Proforma included demographic details, clinical signs and symptoms, history and duration of previous similar signs and symptoms, previous attacks of cholecystitis and acute biliary pancreatitis, previous upper gastrointestinal (GI) surgery, history of endoscopic retrograde cholangiopancreatography (ERCP) with or without common bile duct (CBD) stenting, white blood cell (WBC) count, alkaline phosphatase (ALP), alanine aminotransferase (ALT), total bilirubin, amylase, and lipase. Ultrasound findings such as gallbladder wall thickness (normal or increased, greater than 3 mm), the number and size of stones, presence of sludge, and pericholecystic or peripancreatic collection were documented, along with patient comorbidities. After confirming readiness, general anesthesia was administered, and aseptic drilling was performed. Four ports, including 11 mm umbilical and epigastric ports, were placed. The cystic duct and artery were ligated and cut, facilitating gallbladder retrieval. A drain was placed in the subhepatic space. All patients smoothly recovered from anesthesia and were transferred to postoperative care. Intraoperative findings, including the presence or absence of adhesions (categorized as no adhesions, soft adhesions with surrounding omentum or other structures, or dense adhesions difficult to remove), collections around the gallbladder bed, gallbladder inflammation, ease of manipulation with instruments, successful laparoscopic completion or conversion to open surgery, and duration of the surgery, were recorded.

Inclusion criteria

Included were patients aged 18 years and above and with asymptomatic gallbladder stones, mild-grade acute calculous cholecystitis according to Tokyo guidelines [5], chronic calculous cholecystitis, and gallbladder perforation, irrespective of gender and ethnicity.

Exclusion criteria

Patients with CBD stones, a preoperative diagnosis that required direct open surgery, acalculous cholecystitis, gallbladder malignancy, or acute pancreatitis or who were pregnant or also having procedures like a laparoscopic gastrectomy, Whipple procedure, splenectomy, or ovarian cystectomy were not eligible.

Statistical analysis

Statistical analysis was performed using IBM SPSS Statistics for Windows, Version 23.0 (Released 2015; IBM Corp., Armonk, New York, United States). The chi-squared test, odds ratio (OD), and confidence interval (CI) were calculated for the strength of associations. Statistical significance was established as a p-value less than 0.05. We performed both univariate and multivariate analyses and developed a risk stratification model using exploratory factor analysis.

The characteristics of the two groups, LC completed successfully (LC group) and conversion group (OC group), were compared using the chi-squared test (categorical variables indicated by frequencies and percentages) and Mann-Whitney U test (quantitative variables indicated by mean, standard deviation, median, and ranges). Furthermore, the Shapiro-Wilk test for continuous variables was done for normality.

Univariate logistic regression was done to measure the association of the aforementioned parameters with the rate of conversion to open surgery. Then multivariate analysis was devised to express the likelihood of a particular occurrence as a composite function of various variables. By utilizing the risk factors that exhibited a significant association with conversion, a hierarchical exploratory analysis was done to uncover the pathway for the summation of these risk factors. The predictive quality of conversion to open surgery was assessed by measuring sensitivity and specificity which should be ideally 100%. In addition, the area under the curve (AUC) value and receiver operating characteristic (ROC) curve were noted for the preoperative variable based on logistic regression.

## Results

In this study, 349 patients underwent LC, with 336 (96.3%) success and 13 (3.7%) converted to open surgery, consistent with the lower end of the range in the literature. In 170 patients (50.4%), a simple cholecystectomy was done, while 167 patients (49.6%) underwent a difficult cholecystectomy. The majority of patients were women (75%), and the conversion rate was higher in rural areas (1.42%) compared to urban areas (2.27%). Various factors were analyzed in the study population. The age distribution in the study was non-normal, with a median age of 51 years (IQR=24), with the 25th percentile at 36 years and the 75th percentile at 60 years. The patients in the OC group were significantly older (p=0.022), and there was a higher proportion of men (p=0.019). The OC group also had a higher proportion of patients over the age of 60, but two patients had lower ages (33 and 36 years) due to anatomic anomalies and difficult Calot's anatomy leading to conversion.

Table 1 shows that the LC group included 78 (23.1%) male and 259 (73.6%) female patients, whereas the OC group had seven (2%) male and six (1.6%) female patients.

**Table 1:** Comparison of gender with OC and LC OC: open cholecystectomy; LC: laparoscopic cholecystectomy

Gender	Completed laparoscopically n (%)	Conversion to open n (%)	Total	P-value of group
Male	78 (91.76%)	7 (8.2%)	84 (24.4%)	-
Female	259 (97.7%)	6 (2.29%)	265 (75.6%)	-
Total	337 (96.3%)	13 (3.7%)	349	0.019

Table 2 revealed several significant factors. Symptomatic patients had a higher OD of 1.05 (CI=1.023-1.078; p=0.039), and those who experienced fever had a higher OD of 1.09 (CI=1.04-1.149; p=0.001). Previous attacks of symptoms had a significantly higher OD of 6.2 (CI=3.0-4.08; p=0.012), while a history of jaundice due to gallbladder disease had a significantly higher OD of 26.8 (CI=7.7-93; p=0.001). Past history of pancreatitis due to gallbladder stones (OD=4.0; CI=1.27-12.56; p=0.011), past history of acute calculous cholecystitis (OD=14.3; CI=5.03-41.15; p=0.001), and diabetes (OD=14.7; CI=1.9-114; p=0.01) were also found to be statistically significant risk factors. However, right hypochondrial pain, postprandial pain, past history of GI surgery, hypertension, and other comorbidities were not significant predictors of conversion to OC.

**Table 2:** Outcomes and risk factors in laparoscopic vs. open conversion surgeries RHC: right hypochondrium; Hx: history; GB: gallbladder; sx: surgery; ERCP: endoscopic retrograde cholangiopancreatography; GI: gastrointestinal

Variables	Sub-features	Completed laparoscopically n (%)	Conversion to open n (%)	Risk estimates	Confidence interval	P-value
Gender	Male	78 (23.1%)	7 (2.1%)	3.85	1.26-11.82	0.012
	Female	259 (76.9%)	6 (1.8%)	-	-	-
Symptomatic	Yes	260 (77.4%)	13 (3.9%)	1.05	1.023-1.078	0.039
	No	76 (22.6%)	0 (0%)	-	-	-
RHC pain	Yes	291 (86.6%)	13 (3.9%)	1.045	1.02-1.07	0.157
	No	45 (13.4%)	0 (0%)	-	-	-
Postprandial pain	Yes	309 (91.9%)	13 (3.9%)	1.02	1.04-1.06	0.280
	No	27 (8.1%)	0 (0%)	-	-	-
Fever	Yes	198 (58.9%)	13 (3.9%)	1.09	1.04-1.149	0.001
	No	138 (41.1%)	0 (0%)	-	-	-
Previous attacks	Yes	193 (57.4%)	12 (3.6%)	6.2	3.0-4.08	0.012
	No	43 (12.8%)	1 (0.3%)	-	-	-
Hx of jaundice	Yes	26 (7.7%)	9 (2.7%)	26.8	7.7-93.0	0.0001
	No	210 (62.4%)	4 (1.2%)	-	-	-
Hx of pancreatitis	Yes	96 (28.6%)	8 (2.4%)	4.00	1.27-12.56	0.011
	No	140 (41.7%)	5 (1.5%)	-	-	-
Hx of cholecystitis	Yes	47 (14%)	10 (3%)	14.3	5.03-41.15	0.0001
	No	189 (56.2%)	3 (0.9%)	-	-	-
Hx of surgery	Upper GI surgery	7 (2.1%)	0 (0%)	1.505	0.471-4.82	0.471
	Others	28 (8.3%)	0 (0%)	0.899	0.245-3.30	0.246
	No surgery history	312 (89.6%)	0 (0%)	-	-	-
Hx of ERCP	Yes	13 (3.9%)	6 (1.8%)	1.30	0.98-1.88	0.051
	No	323 (96.1%)	0 (0%)	-	-	-
Comorbidities	Diabetes	151 (43.4%)	12 (3.6%)	14.7	1.9-114.0	0.010
	Hypertension	114 (32.8%)	7 (2.1%)	1.6	0.58-5.08	0.251
	Others	98 (28.2%)	6 (1.8%)	-	-	-

The laboratory parameter analysis (as shown in Table 3) revealed that the WBC count was greater than 10k/mm^3^ (OD=3.7; CI=1.005-12.174; p=0.001), which was a significant finding, while there were no significant differences observed in other parameters between the groups under study.

**Table 3:** Laboratory parameters Deranged white blood cell count: >10,000/µL; deranged alkaline phosphatase: >120 IU/L; deranged alanine transaminase: >45 IU/L; deranged total bilirubin: >1.2 mg/dL x: cannot be computed

Subgroup variables	Values	Completed laparoscopically n (%)	Conversion to open n (%)	Risk estimate	Confidence interval	P-value
White blood cell count (/microliters)	<10	199 (99.01%)	2 (0.99%)	0.01	x	-
	>10	137 (92.5%)	11 (7.46%)	3.7	1.005-12.174	0.001
Alkaline phosphatase	Deranged	67 (98.53%)	1 (1.47%)	0.2	x	0.67
Alanine transaminase	Deranged	32 (91.43%)	3 (8.67%)	1.23	x	0.472
Total bilirubin	Deranged	8 (88.89%)	1 (11.11%)	2.2	x	0.99

Ultrasonographic features show that wall thickness greater than 3 mm (OD=4.28; CI=1.16-15.6; p=0.018), presence of sludge in the gallbladder (OD=1.08; CI=0.12-1.2; p=0.035), and presence of pericholecystic collection (OD=2.04; CI=0.609-6.86; p=0.011) were statistically significant for conversion to open (Table 4).

**Table 4:** Ultrasound findings and their association with laparoscopic vs. open conversion surgeries

Subgroups	Variables	Completed laparoscopically n (%)	Converted to open n (%)	Risk estimate	Confidence interval	P-value
Gallbladder appearance	Distended or contracted	295 (87.3%)	11 (3.3%)	0.76	0.164-3.572	0.733
	Normal	41 (12.1%)	2 (0.6%)	-	-	-
Wall thickness	>3 mm (thickened)	147 (43.5%)	10 (3%)	4.28	1.16-15.6	0.018
	<3 mm (normal)	189 (55.9%)	3 (0.9%)	-	-	-
Sludge presence	Yes	244 (72.2%)	12 (3.6%)	1.08	0.12-1.2	0.035
	No	92 (27.2%)	1 (0.3%)	0.81	0.11-0.3	0.770
Gallbladder stone size	Small stones (>7 mm)	204 (60.3%)	13 (3.8%)	0.996	0.309-3.016	0.950
	Large stones	132 (96.35%)	132 (96.35%)	-	-	-
Number of gallbladder stones	Single	16 (88.8%)	16 (88.8%)	0.27	0.056-1.346	0.090
	Multiple	320 (96.67%)	320 (96.67%)	-	-	-
Pericholecystic collection	Present	60 (17.7%)	4 (1.2%)	2.04	0.609-6.86	0.011
	Absent	276 (81.7%)	8 (2.4%)	1.90	0.55-0.45	0.050

Table 5 summarizes that the presence of gallbladder bed collection (OD=12.7; CI=2.78-58.78; p=0.000), dense adhesions (OD=3.65; CI=0.785-17.028; p=0.018), and difficult gallbladder manipulation with instruments (OD=13.5; CI=1.7-107; p=0.001) were significantly associated with an increased risk of conversion to open surgery. The presence of inflamed gallbladder (OD=4.12; CI=0.53-32.2; p=0.089), anatomic anomaly (p=0.982), and CBD stones (p=0.889) were not found to be significant predictors.

**Table 5:** Intraoperative features x: cannot be computed

Intraoperative findings	Sub-features			Completed laparoscopically n (%)	Conversion to open n (%)	Confidence interval	P-value
Inflamed gallbladder	Yes		250 (95.45%)	12 (4.55%)	4.12	0.53-32.2	0.089
Gallbladder bed collection		Present	101 (90.1%)	11 (9.9%)	12.7	2.78-58.78	0.000
Adhesions	Soft adhesions		85 (100%)	0 (0%)	0.624	0.056-6.994	0.127
	Dense adhesions		145 (92.57%)	11 (7.43%)	3.65	0.785-17.028	0.018
Anatomic anomaly	Yes		2 (33.33%)	4 (66.7%)	2.1	x	0.982
Common bile duct stones	Yes		8 (100%)	0 (0%)	x	x	0.889
Gallbladder manipulation with instruments	Difficult		156 (92.31%)	12 (7.7%)	13.5	1.7-107	0.001

Table 6 summarizes the univariate analysis of all independent variables and multivariate analysis of variables that were significant in the univariate analysis, such as male gender, history of jaundice, pancreatitis, >3 mm wall thickness, WBC >10, difficult to handle with instruments, and dense adhesions.

**Table 6:** Univariate and multivariate logistic regression analyses of variables OC: open cholecystectomy; LC: laparoscopic cholecystectomy; Hx: history; x: cannot be computed

Groups	OC n (%)	LC n (%)	P-value of univariate analysis	Confidence interval	P-value of multivariate analysis	Confidence interval
Age >60 years	5 (5.49%)	86 (94.5%)	0.049	0.89-1.65	0.53	0.003-0.98
Male	7 (8.5%)	78 (91.7%)	0.000	1.260-11.821	0.017	0.679-8.625
Symptomatic	13 (4.76%)	260 (95.3%)	0.997	0.94-1.09	-	-
Fever	13 (8.7%)	138 (91.3%)	0.994	0.97-0.998	-	-
Hx of jaundice	9 (25.7%)	26 (74.3%)	0.000	3.944-50.353	0.000	5.265-34.850
Hx of acute cholecystitis	8 (14.8%)	46 (85.1%)	0.034	1.107-12.231	0.238	0.538-12.219
Hx of pancreatitis	8 (7.8%)	96 (92.3%)	0.047	0.289-3.269	0.023	0.028-1.990
Diabetes	12 (7.9%)	151 (92.6%)	0.011	1.890-114.351	0.414	0.273-23.527
>3 mm wall thickness	10 (6.42%)	147 (93.58%)	0.029	1.159-15.853	0.035	0.010-0.847
Pericholecystic fluids in the gallbladder bed	4 (6.25%)	60 (93.75%)	0.247	0.146-1.641	-	-
Sludge	12 (11.6%)	91 (88.34%)	0.154	0.029-1.750	-	-
White blood cell count >10k	11 (7.46%)	137 (92.5%)	0.001	2.330-25.605	0.012	0.627-29.706
Intraoperative gallbladder inflammation	12 (4.55%)	250 (95.45%)	0.176	0.031-1.891	-	-
Difficult to handle with instruments (thick-walled)	12 (7.7%)	156 (92.3%)	0.012	1.780-107.688	0.046	x
Dense adhesions	10 (7.43%)	145 (92.5%)	0.0091	0.021-1.356	0.03	x

Table 6 shows a graphic, comprehensive presentation of our risk factor summation pyramid, which is 84.6% sensitive to predict the conversion of surgery. The total cumulative risk for conversion to open surgery, combining all these variables, is 84.61%. This summarizes the risk assessment for the likelihood of conversion to open surgery based on these variables. A patient with age >60 years (60 and above)+male+history of cholecystitis or pancreatitis+gallbladder wall thickness greater than 3 mm has a sensitivity of 84.61% for conversion to open; the contributing factor is gallbladder wall thickness greater than 3 mm. This also shows the influence of each risk factor individually. It also shows exploratory pyramidal analysis of the final factors and summation with the highest sensitivity of 84.6% and specificity of 63.8%. The early risk prediction model was made by forward stepwise regression, just selecting significant risk factors into account. This order was chosen to facilitate a clear and intuitive understanding of how various patient characteristics contribute to the overall risk of conversion.

**Table 7:** Risk factor summation pyramid OC: open cholecystectomy; n: number

Risk stratification	Variables	OC % (n)	Summative risk for conversion
1	Age >60 years	38.46% (5)	38.46%
2	Male+first above	23.08% (3)	61.54%
3	Second+history of cholecystitis or acute pancreatitis	15.38% (2)	76.92%
4	Third+gallbladder wall thickness >3 mm	7.69% (1)	84.61%
	Total	84.61%	

**Figure 1:**
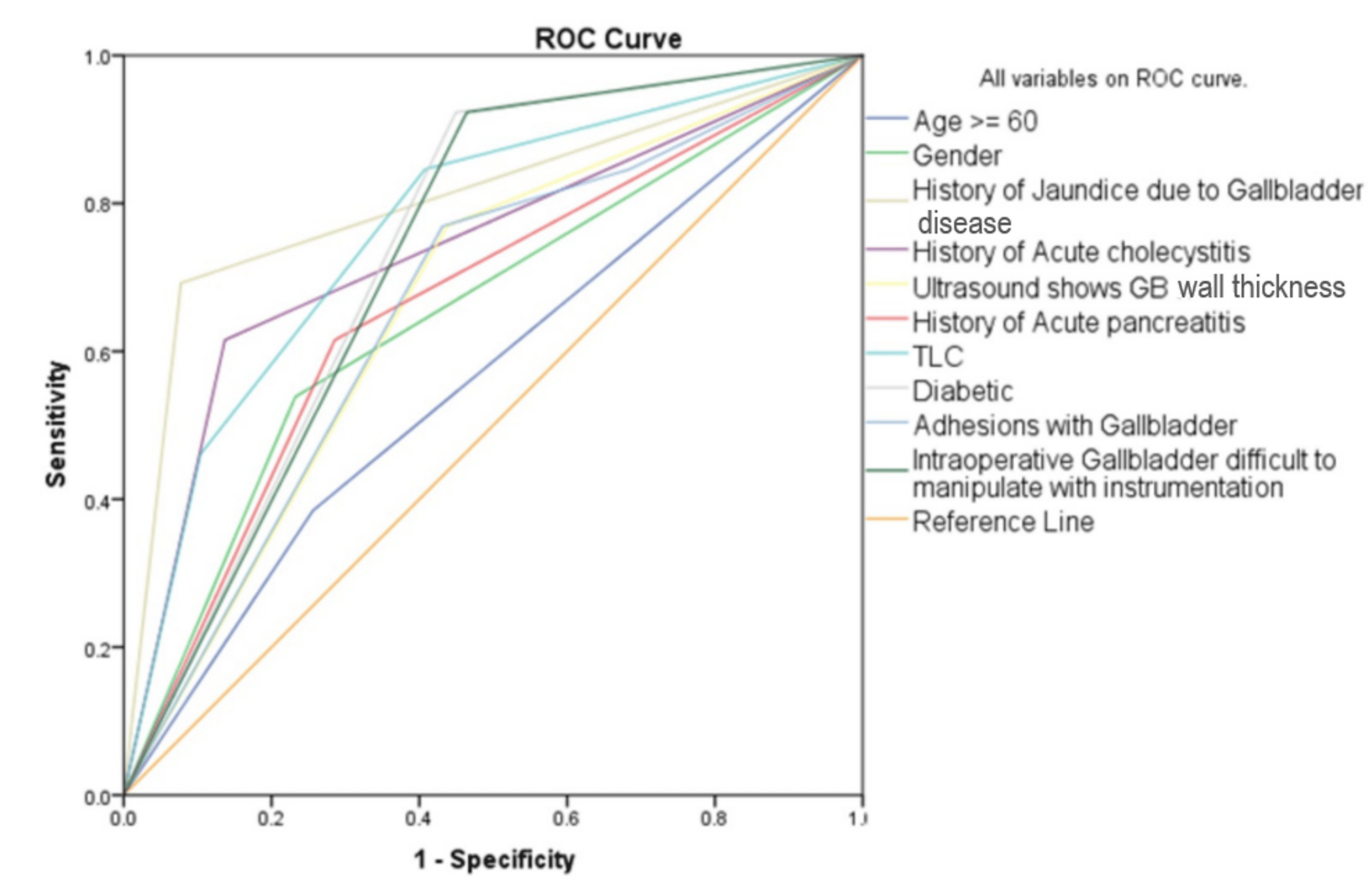
ROC curve of variables ROC: receiver operating characteristic; TLC: total leukocyte count

Figure 1 summarizes the prediction of conversion to open surgery incidence for all the factors in a single curve. The ROC curve indicates good discrimination ability, with an AUC of 0.839, indicating acceptable classification accuracy. The AUC was 0.839 with a sensitivity of 0.769. The regression model has provided diagnostic test quality measures that are considered satisfactory (76.9% sensitivity).

**Figure 2:**
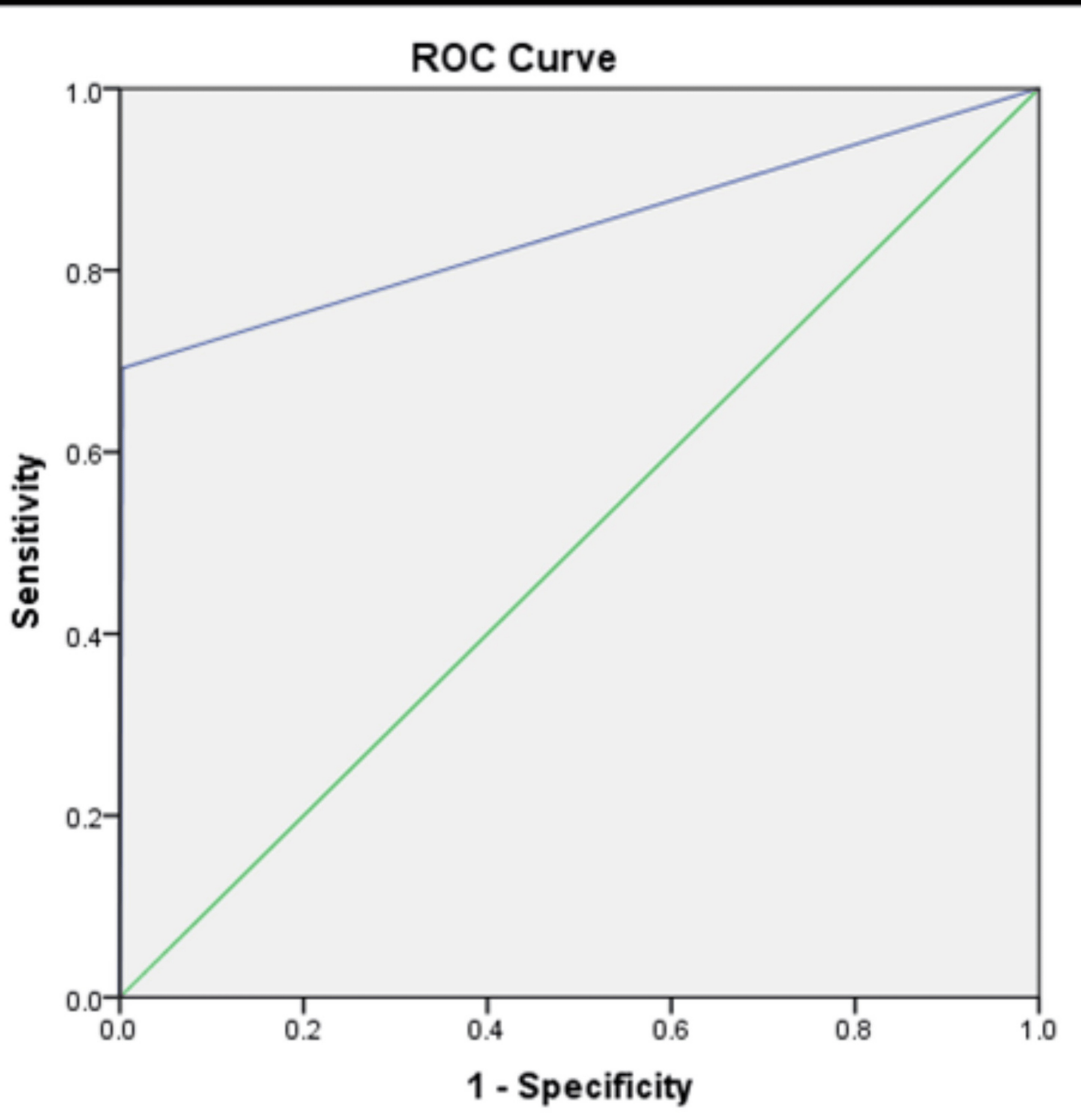
ROC curve of discriminatory performance in predicting the conversion to open surgery ROC: receiver operating characteristic

Figure 2 displays the ROC curves for each factor, providing a detailed overview of their discriminatory performance in predicting the conversion to open surgery.

## Discussion

LC has been established as the preferred and dependable approach for managing benign gallbladder conditions, particularly in patients with symptomatic gallstones, due to its safety and efficacy [1,7,8,15]. However, despite these advantages, some patients require conversion to an open procedure, with the proportion ranging from 2% to 15% across different studies. Identifying the risk factors prior to surgery is crucial to ensuring the optimal outcome and benefit for the patient [13].

In this study, a total of 349 patients were qualified for LC in a period of one year, and the conversion rate was 3.7%. Literature data shows 2-15% of conversion from LC to OC due to various risk factors [9,13]. In a study by Amin et al., the conversion rate was 7.78% [2]. This rate is decreasing with frequently invented procedures of minimal invasiveness and better outcomes, as mentioned by Morales-Maza et al. [12].

After conducting multivariate analysis, the risk factors for conversion that we considered include age, male gender, history of jaundice due to gallbladder disease, history of acute cholecystitis, ultrasound-measured gallbladder wall thickness greater than 3 mm, history of acute pancreatitis, total leukocyte count (TLC), presence of dense adhesions, and intraoperative difficulty in manipulating the gallbladder with instrumentation. The age of 60 years and above was a significant risk factor, which was also reported by Tayeb et al. [16]. However, two patients with lower ages (33 and 36 years) required conversion due to anatomic anomalies and difficult Calot's anatomy. Similarly, in the conversion group, a high proportion of patients were older, as reported by Raman et al. [17]. Age as a risk factor may be related to a longer history of gallbladder disease, masked symptoms, and patient delay, as speculated by Simopoulos et al. and Wevers et al. [18,19].

Our findings suggest that being male is a significant risk factor for conversion, consistent with findings from other studies by Warchałowski et al., Chand et al., and Mok et al. [9,11,20]. This trend has been observed in various types of surgeries by Papandria et al. [21]. Male patients who underwent LC in light of our research had a longer history of symptoms, difficult Calot's anatomy, dense adhesions, and inflamed gallbladder compared to female patients, which is consistent with the findings of Hu et al. [13].

A history of jaundice due to gallbladder disease and a history of calculous cholecystitis were identified as risk factors for conversion based on our observations, which is consistent with the findings of Chand et al., Kama et al., Nassar et al., Nachnani et al., and Beksac et al. [11,22-25]. However, this is in contrast to the study conducted by Mok et al. [20]. Additionally, a history of acute pancreatitis without indicating its severity was also identified as a preoperative risk factor for conversion due to dense peritoneal adhesions, difficulty in manipulating the gallbladder with instrumentation, and episodes of peritonitis, which is also supported by Beksac et al. but not by Vaccari et al. [25,26].

The ultrasound examination showed that gallbladder wall thickness greater than 3 mm and pericholecystic fluid collection were significant in univariate analysis, but only gallbladder wall thickness was correlated with conversion to OC in multivariate analysis, similar to a study by Morales-Maza et al., Siddiqui et al., and Ramírez-Giraldo et al. [12,27,28]. A separate study is needed to explore the correlation between gallbladder wall thickness and conversion, as done by Raman et al. [17]. Multiple gallbladder stones and sizes of stones were not significant, consistent with Siddiqui et al. [27]. Having a TLC greater than 10k has been identified as a risk factor for conversion, which is in line with the results of studies carried out by Amin et al. and Mok et al. [2,20]. However, a meta-analysis conducted by Philip Rothman et al. found no association with conversion [29].

According to our results, there was no observed association between ALP, ALT, total bilirubin, lipase, and amylase levels with conversion to OC, but they did show a correlation with surgical difficulty, as noted in the study by Chand et al. [11]. Pancreatic enzymes were not found to be associated with conversion but with difficulty in surgery, and there is no other known study to support this study except for the research conducted by Di Buono et al. [8]. This study is in contrast with the study by Tayeb et al. and Beksac et al. [16,25]. This study shows that diabetes is a risk factor for conversion to OC, which is consistent with the findings of previous studies such as Amin et al., Terho et al., and Ibrahim et al. [2,30,31]. However, the study conducted by Philip Rothman et al. did not support this correlation [29].

Our investigation did not reveal any association between cardiovascular disease and the conversion to OC, which is not consistent with the available literature that we are aware of. However, our results contradict the findings of Vaccari et al. [26].

Our results underscore that a history of previous abdominal surgery was not found to be a significant risk factor for conversion, which is not consistent with the existing literature. The presence of intra-abdominal adhesions due to previous surgery can make the surgery difficult for the surgeon and is a frequent cause of readmissions in hospitals. Several studies, including Amin et al., Nachnani et al., Beksac et al., Ibrahim et al., Tufo et al., and Jang et al., have reported this association [2,24,25,31-33]. Based on our observations, all patients with a history of upper GI surgery underwent LC successfully, but they did have dense adhesions and a longer duration of surgery.

In the context of this study, we did not find any significant correlation between preoperative ERCP with or without CBD stenting and the occurrence of OC. Our findings are in agreement with studies by Amin et al. and Ramírez-Giraldo et al. [2,28]. However, it was in contrast to the results reported by Vaccari et al. [26].

During the intraoperative period, we consider dense adhesions and difficulty in manipulating the gallbladder with instruments as risk factors for conversion. These factors are associated with a longer duration of surgery, tearing of the gallbladder, and spending more time dissecting gallbladder adhesions and Calot's triangle. Dissecting Calot's triangle may take longer in patients with difficult manipulation, inflammation, and dense adhesions, although this may vary depending on the surgeon's skill level and experience. Difficult cholecystectomy was evaluated on the basis of preoperative features, ultrasound findings, and intraoperative findings as discussed in multiple studies [27,28,34-41].

We did not find a significant correlation between inflammation around the gallbladder and conversion to OC. However, when inflammation or adhesions are present, it is important to perform careful dissection during LC. Surgeons should be aware of the increased risk and take appropriate measures to mitigate it, such as carefully dissecting adhesions or switching to open surgery if necessary. This conclusion is supported by studies conducted by Yajima et al. and Sugrue et al. [35,42].

After conducting a logistic regression analysis, we identified the factors that increase the risk of conversion to OC in our study. We used this analysis to develop an early prediction model for risk management during LC. Several other studies in this field have also proposed predictive models based on specific risk factors to estimate the likelihood of conversion from LC to OC, such as those proposed by Warchałowski et al. and Ibrahim et al. [9,31].

From our perspective, we identified a new factor that has a significant contribution to the risk of conversion to OC, namely, technical difficulty in manipulating the gallbladder with instruments. The difficulty in gallbladder manipulation can be influenced by a range of variables, including the patient's gallbladder condition (e.g., thick wall, impacted stones, contracted or distended gallbladder), the surgeon's skill level, and the type of instruments used. We recognize that these factors are essential considerations in the assessment of gallbladder manipulation.

Limitations

This study's limitations included an observational study design pattern and a limited sample size of OC. The sampling size was non-probability consecutive. Cholecystectomies were performed by multiple consultant general surgeons, and it may introduce potential bias. We recommend a study of a larger cohort of OC with a better study design. Lastly, it was a single-center study.

## Conclusions

The conversion rate from LC to OC was 3.7% in our population. The study found several risk factors associated with the possibility of converting LC to open surgery. Clinicians should be aware of the identified risk factors to optimize decision-making and improve surgical outcomes in patients undergoing LC. By understanding and addressing these risk factors, healthcare providers can enhance patient care, reduce complications, and ultimately improve the overall success rate of LC procedures. Moreover, incorporating these insights into preoperative evaluations and surgical planning can help in better anticipating potential challenges during LC, leading to more efficient and safer surgical practices.
